# The Pocket Anatomist

**Published:** 1891-09

**Authors:** 


### BOOK NOTICES.

The Pocket Anatomist. Founded upon Gray. By C. Henri Leonard, A. M., M. D., Professor of the Medical and Surgical Diseases of Women and Clinical Gynæcology, in the Detroit College of Medicine. Fourteenth revised edition, containing Dissection Hints and Visceral Anatomy. Detroit, Mich., 1891. The Illustrated Medical Journal Co., Publishers. Cloth, 297 pages, 193 Illustrations. Price, postpaid, $1.00.

This book is issued on thin, though nicely glazed paper, and takes up but little room, though 300 pages in thickness. The plates introduced are photo-engraved from the English edition of Gray, and are therefore exact; most of them are full-paged, and where they are not, they are grouped together so as to save as much thumbing as possible. The useless "questions" are absent in this work, and their room given to needed illustrations or terse descriptions of the minor parts found in the several dissections made. The chapter given to "dissection hints" gives the lines of incision necessary to best expose the underlying organs, arteries, nerves, or muscles. The chapter on Gynæcological Anatomy can be found only in the more expensive work of Savage. The pronunciation of each anatomical term is given, be it artery, vein, nerve, muscle, or bone. Over 100 pages are devoted to the anatomy of the special organs and viscera. The book has been honored by a re-printing in England after some three thousand copies had been sold over there by the American publishers.

